# Transplantation of Hypoxic-Preconditioned Bone Mesenchymal Stem Cells Retards Intervertebral Disc Degeneration via Enhancing Implanted Cell Survival and Migration in Rats

**DOI:** 10.1155/2018/7564159

**Published:** 2018-02-14

**Authors:** Weiheng Wang, Yang Wang, Guoying Deng, Jun Ma, Xiaodong Huang, Jiangming Yu, Yanhai Xi, Xiaojian Ye

**Affiliations:** ^1^Department of Orthopaedics, Changzheng Hospital, Second Military Medical University, Shanghai 200003, China; ^2^Department of Orthopaedics, Nanjing General Hospital, Nanjing 210000, China; ^3^Trauma Center, Shanghai General Hospital, Shanghai Jiao Tong University School of Medicine, Shanghai 201620, China

## Abstract

**Objective:**

Special hypoxic and hypertonic microenvironment in intervertebral discs (IVDs) decreases the treatment effect of cell transplantation. We investigated the hypothesis that hypoxic preconditioning (HP) could improve the therapeutic effect of bone mesenchymal stem cells (BMSCs) to IVD degeneration.

**Methods:**

BMSCs from green fluorescent protein-transgenic rats were pretreated with cobalt chloride (CoCl_2_, 100 *μ*M, 24 h) for hypoxic conditions *in vitro*. Apoptosis (related pathways of caspase-3 and bcl-2) and migration (related pathways of HIF-1*α* and CXCR4) were detected in BMSCs. *In vivo*, BMSCs and HP BMSCs (H-BMSCs) were injected into the rat model of IVD degeneration. The IVD height, survival, migration, and differentiation of transplanted BMSCs and matrix protein expression (collagen II, aggrecan, and MMP-13) were tested.

**Results:**

H-BMSCs could extensively decrease IVD degeneration by increasing IVD height and collagen II and aggrecan expressions when compared with BMSCs. Significantly, more GFP-positive BMSCs were observed in the nucleus pulposus and annulus fibrosus regions of IVD. HP could significantly decrease BMSC apoptosis (activating bcl-2 and inhibiting caspase-3) and improve BMSC migration (increasing HIF-1*α* and CXCR4) *in vitro*.

**Conclusion:**

HP could significantly enhance the capacity of BMSCs to repair DDD by increasing the survival and migration of implanted cells and increasing matrix protein expression.

## 1. Introduction

According to statistics, 70% of the population will be plagued by lower back pain in life caused by disc degeneration disease (DDD), which leads to a serious public health problem [1–3]. Surgery may be able to alleviate clinical symptoms, although it is unable to eliminate the degeneration of intervertebral discs (IVDs) [4, 5]. Disc degeneration is characterized by a reduction of nucleus pulposus cells (NPCs) and their extracellular matrix (ECM), and NPCs are replaced by cells of a more fibroblast-like phenotype in the degenerated IVDs [6]. Curative treatment for DDD is a major challenge because of limited regeneration of the IVD itself.

Cell transplantation has emerged as an effective therapy for DDD in recent decades [7]. Bone mesenchymal stem cells (BMSCs) hold great promise in IVD regeneration [8–11]. BMSCs are pluripotent and multipotent and possess the capabilities of self-renewal and differentiation into specific cell lineages [12–16]. However, there are hurdles that need to be removed before BMSC transplantation for clinical applications. IVD is the largest avascular tissue in the human body, and nutrition renewal can only be completed through passive diffusion [17, 18]. Due to the lack of blood vessels and the special hypertonic and hypoxic environment of IVDs [19, 20], the survival rate and repair ability of transplanted stem cells cultured under common culture conditions are poor [21, 22]. As the hypertonic and hypoxic environment can reduce activity and vitality of transplanted stem cells [23, 24], a way to bolster the transplanted stem cell survival and treatment effects is needed urgently.

Hypoxic preconditioning (HP) is a powerful, endogenous protective mechanism, which can enhance cell tolerance to subsequent injury and therapy ability [25]. Some studies have shown that HP can effectively improve the transplanted stem cell survival and treatment in myocardial infarction [26] and cerebral infarction [27, 28] animal models. The possible mechanisms of time- and concentration-dependent HP include regulating intracellular transduction, increasing cell resistance to injury, upregulating migration and differentiation, and enhancing growth factor secretion [28–32]. Given the benefits of HP and the special hypertonic and hypoxic environment of IVDs, we supposed the hypothesis that HP might be a feasible and effective way to improve the therapeutic effect of BMSCs to retard IVD degeneration via enhancing implanted cell survival and migration in rats.

The HP conditions were confirmed by a gradient experiment before transplantation, and then the changes of the hypoxic signal pathway, apoptosis and migration ability, and related molecular mechanism in BMSCs were detected *in vitro*. BMSCs and HP BMSCs (H-BMSCs) were transplanted into degenerated IVD rat model to verify the role of HP on cell survival and therapeutic effect *in vivo*.

## 2. Materials and Methods

### 2.1. Animals and Establishment of the DDD Model

Sprague-Dawley (SD) rats (males, 3 months old) and green fluorescent protein- (GFP-) transgenic male SD rats (SD-Tg (CAG-EGFP) CZ-004Osb, Sina-British SIPPR/BK Lab, Animal Ltd., China) purchased from the Experimental Animal Center of Second Military Medical University were used in this study. The rats' skeleton reached maturity at 3 months, and the IVD remodeling proved irrelevant to rat growth [33]. All procedures were approved by the Institutional Animal Care and Use Committee. The DDD model was established as described by Rousseau et al. [34]. Briefly, a longitudinal incision, approximately 2 cm, was made along the tail to expose the lateral portion of the tail discs, Co5/Co6 and Co6/Co7, and then a 21-gauge needle was inserted 1.5 mm into the disc to aspirate an identical defined volume of the nucleus pulposus material. Radiographic images were captured to ensure that the needle was parallel to the endplates to avoid endplate damage.

### 2.2. BMSC Culture and Hypoxia Protocol

The extraction and purification of BMSCs from GFP transgenic rats were performed based on a previous study [35]. BMSCs were passaged when cell fusion rate was between 70% and 80%. The purity of 3 generation (P3) BMSCs were identified by CD29 (FITC), CD90 (PE), CD45 (APC), and CD31 (PE, Guge, Nanjing, China) by flow cytometry (FCM, Beckman, CA, USA). Alizarin red and oil red O (Sigma, MO, USA) were used to examine the osteogenic and adipogenic properties of P3 BMSCs. P3 BMSCs were cultured in fresh complete medium and were added to different concentrations of cobalt chloride (CoCl_2_) for 6, 12, 24, and 48 h. The CoCl_2_ concentration in the complete medium was maintained at 0, 10, 50, 100, 200, and 300 *μ*M.

### 2.3. Detection of the Influence of HP on BMSC Proliferation by CCK-8

P3 BMSCs were inoculated into 96-well plates (5 × 10^3^ cells/well with 100 *μ*L of complete culture medium), and after the cells adhered to the walls, different intervention culture mediums were replaced: the complete culture medium for cells containing 0, 10, 50, 100, 200, and 300 *μ*M CoCl_2_ were cultured for 6, 12, 24, and 48 h. The proliferation of BMSCs was detected using 450 nm ultraviolet ray by a microplate reader (Bio Tek, Vermont, USA) by using a CCK-8 kit (Biyuntian, Jishou, China) according to the manufacturer's instructions. The experiment was repeated 3 times with 3 repeated wells per concentration.

### 2.4. Detection of the Influence of HP on BMSC Migration by Transwell Assay

In this experiment, we used polycarbonate inserts (pore size 8.0 *μ*m, Millipore, Massachusetts, USA) to establish a migration model in a transwell system. The transwell method was conducted according to the manufacturer's instructions. The experiment was divided into BMSC group and H-BMSC group. P3 BMSCs were pretreated with complete medium for 24 h in BMSC group, while P3 BMSCs were pretreated with 100 *μ*M CoCl_2_ for 24 h in the H-BMSC group. Then, cells were cultured in a transwell system with complete medium. Transwell inserts were removed after culturing for 6, 12, and 24 h. The cells were washed with 0.01 M phosphate-buffered saline (PBS) 3 times and fixed with 4% paraformaldehyde for 30 min. Then, the migrated BMSCs were stained with 0.1% crystal violet (Guge, Wuhan, China) for 20 min. The images were captured with a microscope (Olympus, Tokyo, Japan). Five horizons were selected randomly for cell counting. The experiment was repeated 3 times with each using 3 repeated wells.

### 2.5. Detection of the Influence of HP on BMSC Tolerance to Apoptotic Insult by FCM

According to the results of cell proliferation, 100 *μ*M CoCl_2_ for 24 h was selected for the HP conditions. P3 BMSCs were inoculated into 6-well plates at a density of 1 × 10^5^ cells/well and cultured for 24 h. The adherent cells were collected, and the apoptosis rate was detected by FCM by using the annexin V-PI kit (BD, New Jersey, USA). Then, we challenged the BMSCs with serum deprivation for 24 h. Adherent cell apoptosis was detected by FCM to study the BMSC tolerance to serum deprivation. All procedures were carried out according to the manufacturer's instructions. The experiment was repeated 3 times with each using 3 repeated wells.

### 2.6. Detection of the Influence of HP on BMSC mRNA Expression by RT-PCR

The influence of HP on the expression of BMSC migration-related genes (HIF-1*α* and CXCR4) and apoptosis-related genes (caspase-3 and bcl-2) was detected. P3 BMSCs were cultured in 6-well plates for 24 h at 0 and 100 *μ*M CoCl_2_. Then, BMSCs were challenged with serum deprivation for 24 h. Total mRNA was extracted using isogen reagent and suggested protocols (Nippon Gene, Tokyo, Japan). RNA samples were then reverse transcribed to cDNA, followed by specific amplification of matrix-specific genes and electrophoretic separation. The mRNA expression of target genes was normalized to that of GAPDH. The experiment was repeated 3 times. The primers were synthesized by Shenggong Biomedical Engineering (Shanghai, China, Table 1).

### 2.7. The Administration of BMSCs

Forty SD rats were randomly divided into 4 groups: the sham group, control group, BMSC group, and H-BMSC group (*n* = 10). In the sham group, incisions were made without inserting a needle. Rats in the control, BMSC, and H-BMSC groups were inserted with a 21-gauge needle. After 2 weeks, cell transplantation into Co5/Co6 and Co6/Co7 discs was performed carefully through a 33-gauge microinjector (Hamilton, Switzerland) for at least 5 min. 2.0 *μ*L PBS (0.01 M) was injected in the control group, while P3 GFP-positive BMSCs (BMSC-GFP, 2 × 10^4^ cells) dissolved in 2.0 *μ*L PBS were injected in the BMSC group and HP P3 BMSC-GFP (2 × 10^4^ cells) dissolved in 2.0 *μ*L PBS were injected in the H-BMSC group. Four weeks after cell transplantation, the IVDs of Co5/Co6 and Co6/Co7 were obtained for the subsequent study.

### 2.8. Radiographic Analysis

At 1 day before and 2, 4, and 6 weeks after surgery, lateral plain radiographs were taken after anesthesia to keep the caudal muscle relaxed. The disc height was calculated by disc height index (DHI) as described by Lin et al. [36]. Changes in DHI were expressed as % DHI and normalized to the measured preoperative IVD height (% DHI = postoperative DHI/preoperative DHI × 100, *n* = 10). Changes in IVD height at different weeks were analyzed by Sante DICOM free software. All images were measured by 3 independent observers who were blind to the specimens.

### 2.9. Evaluation Survival, Migration, and Differentiation of Transplanted BMSCs

Four weeks after cell transplantation, Co6/Co7 discs were harvested and processed individually in Tissue-Tek O.C.T. Compound (*n* = 10). The tissues were sectioned with a freezing microtome (LEICA, Germany) in the coronal direction to generate 7 *μ*m thick sections. Sections were fixed with 4% paraformaldehyde and incubated overnight with rabbit anti-rat collagen II (1 : 200, ab188570, Abcam, USA) and rabbit anti-rat aggrecan (1 : 200, 13880-1-AP, Proteintech, USA) primary antibodies at 4°C. Then, the sections were incubated with the corresponding secondary antibody for 2 h at room temperature. Cell nuclei were stained by 4′,6′-diamino-2-phenylindole (DAPI, HARVEY, USA). The sections were observed by a fluorescence microscope (Olympus, Tokyo, Japan). 10 fields were randomly selected from each group to calculate the cell-positive rate. The rate of positive cells was calculated as (number of positive cells)/(total number of cells in the field) × 100%.

### 2.10. Changes of Nucleus Pulposus Tissue Matrix Protein (Collagen II, Aggrecan, and MMP-13) Detected by Western Blot

Antibodies of rabbit anti-rat collagen II (ab188570, Abcam, USA), rabbit anti-rat aggrecan (13880-1-AP, Proteintech, USA), and rabbit anti-rat MMP-13 (ab39012, Abcam, USA) were used to detect protein expression in nucleus pulposus tissue by Western blot. Four weeks after cell transplantation, Co5/Co6 discs were harvested (*n* = 10), and the total protein in each sample was determined by the BCA method. Cytosolic fractions were separated by SDS-PAGE, transferred, and immobilized on a nitrocellulose membrane. Using corresponding secondary antibody (1 : 15,000; Abmart, Shanghai, China) for 2 h at room temperature, the immune complexes were detected with the ECL chemiluminescence system. Protein from densitometry was quantitatively analyzed with Sigma Scan Pro 5 and normalized to the GAPDH level.

### 2.11. Statistical Analysis

All results are presented as the mean ± SD or mean ± SEM. Data analysis was performed by SPSS 21 software (SPSS Inc., Chicago, USA), and diagrams were drawn by GraphPad Prism 5 software (GraphPad Inc., California, USA). The data was analyzed by repeated measure ANOVA test. *P* < 0.05 was considered statistically significant.

## 3. Results

### 3.1. Characterization of BMSCs

**Figure 1:**
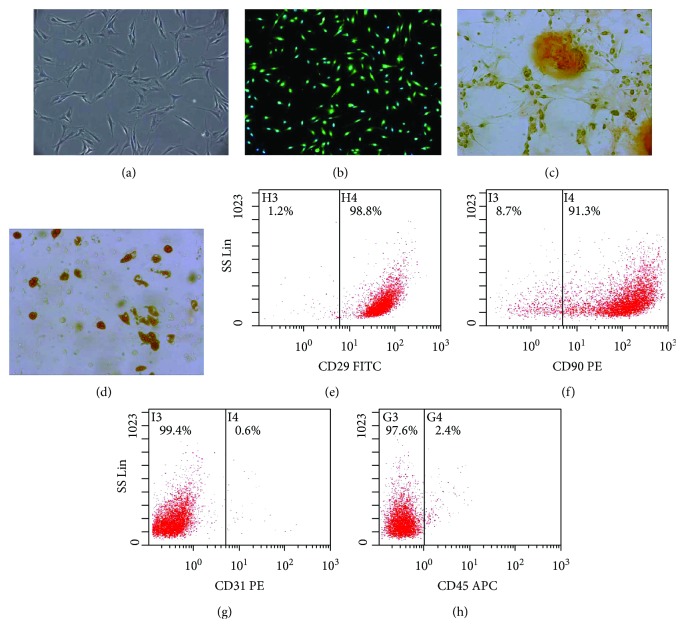
(a) Morphology of BMSCs was observed under microscope (200×). (b) BMSC-GFP displayed green fluorescence and blue nuclei under fluorescence microscopy (100×). (c) Osteogenic differentiation of BMSCs: dark brown calcium nodules stained by alizarin red were observed in cells (400×). (d) Adipogenic differentiation of BMSCs: brown lipid droplets stained by oil red O were observed in cells (400×). (e–h) The P3 BMSC-positive rates of CD29, CD90, CD31, and CD45 were detected by FCM.

Identification of the morphology, purity, and differentiation of P3 BMSCs was performed. P3 BMSCs were uniform, spindle-shaped, or irregularly refractive (Figure 1(a)). Fluorescence microscopy showed that P3 BMSCs were labeled with green fluorescence (Figure 1(b)). BMSC-GFP expression is driven by the chicken *β*-actin promoter and cytomegalovirus enhancer [37] and confirmed to be GFP positive in a previous study [38]. CD29 (+), CD90 (+), CD31 (−), and CD45 (−) were used to detect the purity of P3 BMSC by FCM. The positive rate of CD29, CD90, CD31, and CD45 was 98.8%, 91.3%, 0.6%, and 2.4%, respectively (Figures 1(e)–1(h)), which indicated that P3 BMSCs were of high purity. As shown in Figures 1(c) and 1(d), BMSCs had a good ability for osteogenic and adipogenic differentiation. All of these results suggested that the obtained high-purity BMSCs were suitable for cell therapy.

### 3.2. Influence of HP on BMSC Proliferation

The different concentrations of CoCl_2_ could decrease proliferation, which correlated with the CoCl_2_ concentrations and cell culture time (Table 2). When the CoCl_2_ concentrations were 200 and 300 *μ*M, the BMSC proliferation was significantly inhibited, and the inhibition extent was more obvious with the extension of culture time (*P* < 0.05). According to the results of CCK-8, the condition of 100 *μ*M CoCl_2_ at 24 h was selected as the HP condition. Under this condition, the influence of HP on BMSC proliferation was significant and acceptable.

### 3.3. HP Decreased Cell Apoptosis

**Figure 2:**
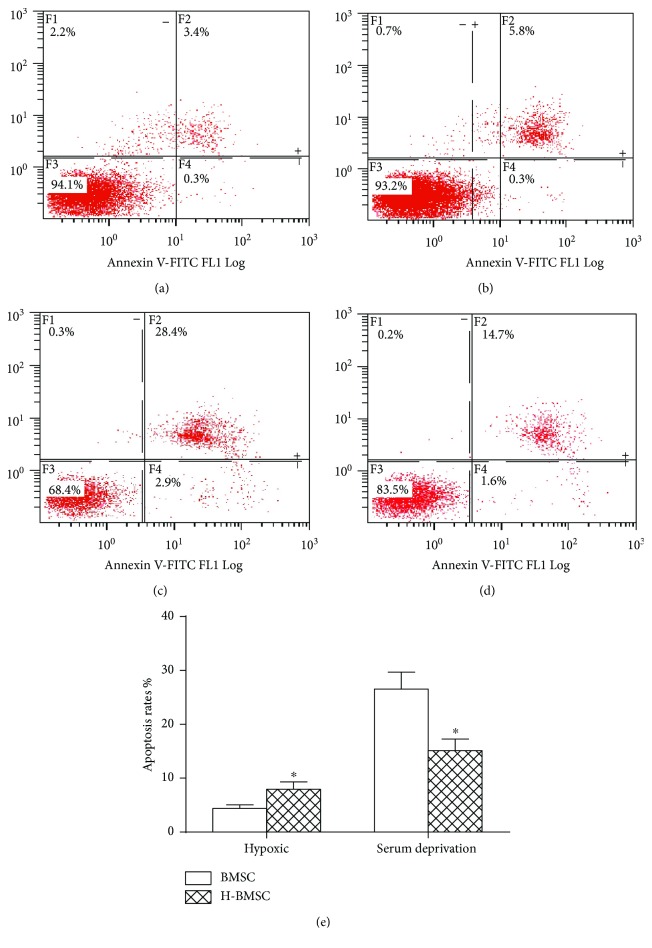
HP increased BMSC tolerance to serum deprivation. (a–d) show the apoptosis rate of BMSCs detected by FCM. (e) shows the apoptosis rate of BMSCs. Data presented here is the mean ± SD. ^∗^ *P* < 0.05 versus BMSC group (*n* = 6).

Apoptosis is a major form of cell death. The apoptosis rate was the early plus late apoptosis rate. FCM detection results were shown after 100 *μ*M CoCl_2_ preconditioned for 24 h; the apoptosis rate of BMSCs was significantly increased (Figures 2(a) and 2(b), *P* < 0.05), while the rate was less than 10%, which was within the acceptable range. Based on cell viability and apoptosis rate results, 100 *μ*M of CaCl_2_ for 24 h was selected as the experimental model of chemical HP of BMSCs. Under this condition, HP had a significant influence on BMSC proliferation and apoptosis rate was within an acceptable range. Then, we challenged BMSCs with serum deprivation for another 24 h. FCM detection results showed that HP could sufficiently decrease the BMSC apoptosis rate and increase BMSC tolerance to serum deprivation (Figures 2(c) and 2(d), *P* < 0.05).

### 3.4. HP Upregulated the Migration of BMSCs

**Figure 3:**
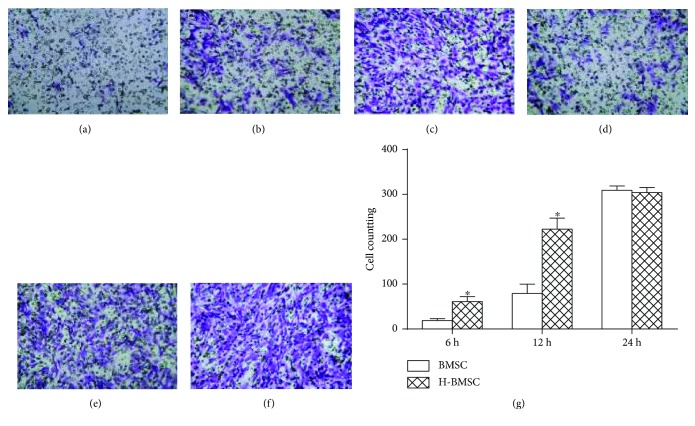
HP upregulated the migration of BMSCs. (a), (b), and (c) show the number of migrated cells in the BMSC group at 6, 12, and 24 h (200×). (d), (e), and (f) represent the number of migrated cells in the H-BMSC group at 6, 12, and 24 h (200×). (g) shows the number of migrated BMSCs in two groups at different times. Data presented here is the mean ± SD.^∗^ *P* < 0.05 versus BMSC group (*n* = 6).

The transwell experiment showed that HP upregulated the migration of BMSCs. There were significantly more cells passing through the membrane in the H-BMSC group after culture for 6 h (Figures 3(a) and 3(d), *P* < 0.05) and 12 h (Figures 3(b) and 3(e), *P* < 0.05) than in the BMSC group. After culture for 24 h, the difference between the two groups was not significant (Figures 3(c) and 3(f), *P* > 0.1), indicating that most of the BMSCs had passed through the membranes in two groups. HP could significantly increase the migration ability of BMSCs.

### 3.5. HP Enhances BMSC Migration via HIF-1*α* and CXCR4 Pathways and Tolerance to Serum Deprivation by Regulating Bcl-2 and Caspase-3

**Figure 4:**
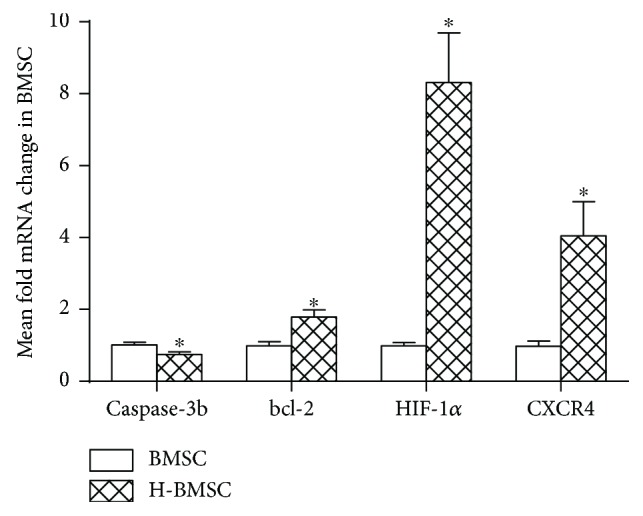
HP enhances BMSC migration via HIF-1*α* and CXCR4 pathways and tolerance to serum deprivation by regulating bcl-2 and caspase-3. Data presented here is the mean ± SD. ^∗^ *P* < 0.05 versus BMSC group (*n* = 6).

The BMSC mRNA expression of caspase-3, bcl-2, HIF-1*α*, and CXCR4 was determined by using RT-PCR. Since caspase-3 and bcl-2 activation is a key pathway of apoptosis, we analyzed the mRNA expression to determine whether HP can decrease apoptosis via this pathway. HIF-1*α* and its downstream gene, CXCR4, are considered key factors in the role of migration and homing. Therefore, we examined the mRNA content to study whether HP can increase BMSC migration via this pathway. Compared with the BMSC group, the mRNA expression of bcl-2, HIF-1*α*, and CXCR4 in the H-BMSC group was significantly increased, while the caspase-3 was significantly decreased (Figure 4, *P* < 0.05). These results indicated that HP could significantly enhance the BMSC migration via HIF-1*α* and CXCR4 pathways and tolerance to apoptotic insult by regulating bcl-2 and caspase-3.

### 3.6. HP Enhances BMSC Ability of Maintaining IVD Height

**Figure 5:**
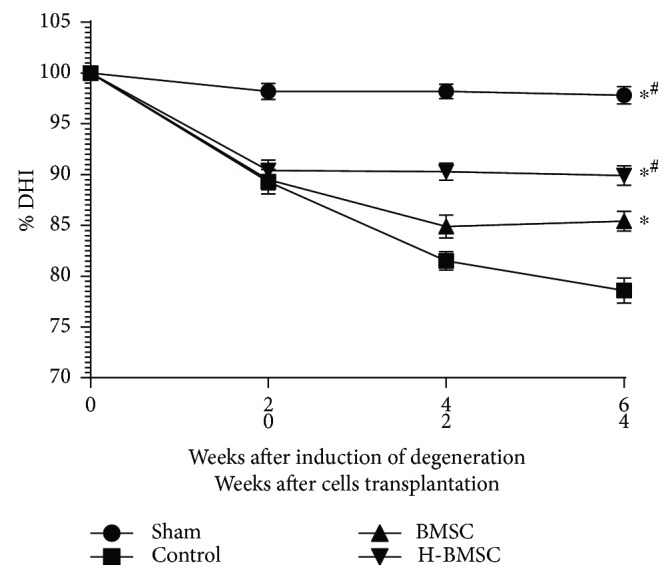
Change of the DHI at different time points after cell transplantation. Data presented here is the mean ± SEM. ^∗^ *P* < 0.05 versus control group, ^#^ *P* < 0.05 versus BMSC group (*n* = 10).

Radiography showed that DHI in the sham group did not change significantly (*P* > 0.1). DHI decreased after the induction of IVD degeneration in the control, BMSC, and H-BMSC groups (Figure 5). The mean DHI in the control group continued to decrease until 6 weeks after the induction of IVD degeneration. DHI in the BMSC and H-BMSC groups was much higher compared with that in the control group (*P* < 0.05), and the mean DHI in the H-BMSC group was much higher than that in the BMSC group at 4 weeks after cell transplantation (*P* < 0.05). All these data indicated that BMSC administration could maintain the IVD height, and HP could enhance this ability.

### 3.7. HP Enhanced BMSC Survival and Migration in Degenerated IVD

**Figure 6:**
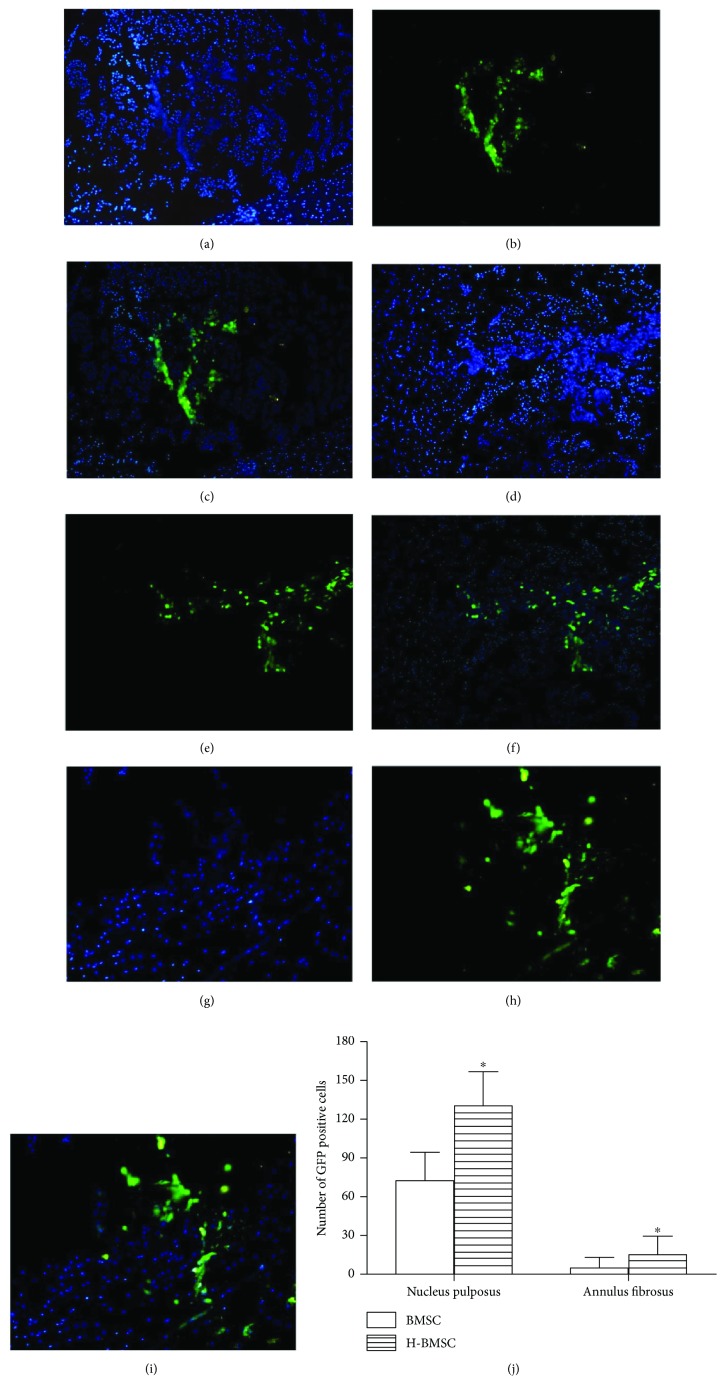
The survival and migration of BMSCs 4 weeks after cell transplantation. (a–c) show the BMSC group, (d–i) show the H-BMSC group. (a), (d), and (g) show the cell nucleus image stained by DAPI. (b), (e), and (h) show GFP-positive cells. (c) Merges of (a) and (b) (100×). (f) Merges of (d) and (e) (100×). (i) Merges of (g) and (h) (400×). (j) shows the number of BMSC-GFP in the nucleus pulposus and annulus fibrosus regions. Data presented here is the mean ± SD.^∗^ *P* < 0.05 versus BMSC group (*n* = 10).

BMSC-GFP were detected in IVD after 4 weeks of cell transplantation (Figure 6). There were no GFP-positive cells in the sham and control groups (data not shown). After cell transplantation, BMSC-GFP were mostly distributed in the center of IVD, the density of cells around the transplanted cells was larger, and there was more ECM around the transplanted cells (Figures 6(b), 6(e), and 6(h)). While in the H-BMSC group, BMSC-GFP were relatively dispersed among the whole IVDs. Some BMSC-GFP migrated into the annulus fibrosus in 6 of the 10 discs in the H-BMSC group (Figures 6(e) and 6(f)), as well as 2 of the 10 in the BMSC group. The number of BMSC-GFP in the nucleus pulposus region and annulus fibrosus region of the H-BMSC group was much higher compared with that of the BMSC group (Figure 6(h), *P* < 0.05). These mean that much more BMSC-GFP in H-BMSC group can migrate into annulus fibrosus region, and HP can upregulate the migration of BMSCs. BMSC-GFP localized in the nucleus pulposus region presented a more rounded shape, similar to the native nucleus pulposus cells (Figures 6(b) and 6(e)). The BMSC-GFP in the inner annulus fibrosus region were spindle-shaped and similar to native annulus fibrosus cells, which may “differentiate” into annulus fibrosus cells (Figures 6(g)–6(i)). From the immunohistochemistry result (supplementary Figure 1), we found that the transplanted BMSCs could express the collagen II and aggrecan. We also found that all of the NP cells and the extracellular matrix in the nucleus pulposus region were stained by immunohistochemistry collagen II and aggrecan, and the transplanted BMSCs surround the positive extracellular matrix. All these dates may lead to the false positive of transplanted BMSCs and need more future study. HP enhanced BMSC survival and migration after transplantation into the degenerated IVD.

### 3.8. HP Enhanced BMSC Matrix-Associated Protein Expression

**Figure 7:**
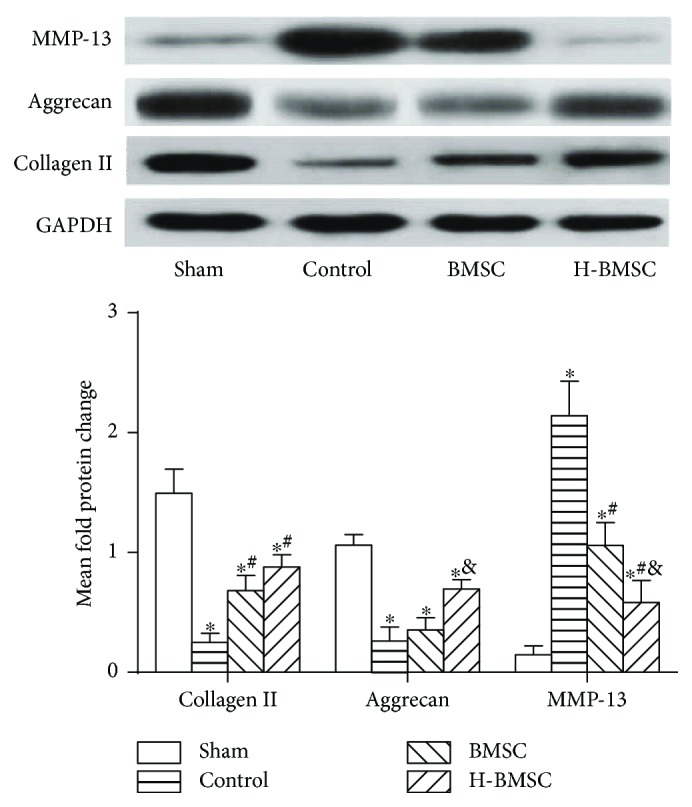
HP enhanced BMSC matrix-associated protein expression (*n* = 6). Data presented here is the mean ± SD. ^∗^ *P* < 0.05 versus sham group; ^#^ *P* < 0.05 versus control group; ^&^ *P* < 0.05 versus BMSC group (*n* = 5).

Western blot was utilized to analyze the expression of collagen II, aggrecan, and MMP-13 protein in nucleus pulposus tissue after 4 weeks of cell transplantation (Figure 7). Collagen II and aggrecan were significantly decreased in the control, BMSC, and H-BMSC groups, compared with the sham group (*P* < 0.01), indicating that matrix-associated protein expression was reduced significantly when the IVDs were degenerated. Meanwhile, the protein expression of collagen II and aggrecan in the H-BMSC group was significantly higher than that in the BMSC group (*P* < 0.05). MMP-13 protein expression increased significantly in the control group compared with sham group (*P* < 0.05). In comparison with the BMSC group, the MMP-13 level decreased drastically in the H-BMSC group (*P* < 0.05). All of these results indicated that BMSCs enhanced matrix associated collagen II and aggrecan protein expression by suppressing the MMP-13 pathway and that HP could upregulate this effect.

## 4. Discussion

The aim of this research was to prove the hypothesis that HP can enhance the capacity of BMSCs to repair DDD by increasing implanted cell survival and migration. Based on the result of BMSC proliferation (Table 2) and apoptosis (Figure 2), pretreatment with 100 *μ*M CoCl_2_ for 24 h was selected as the HP condition in this experiment. We found that HP could significantly enhance the migration ability of BMSCs via pathways of HIF-1*α* and CXCR4 and decrease BMSC apoptosis by the pathways of caspase-3 and bcl-2 *in vitro* (Figure 4). The effect of HP on BMSC migration and apoptosis *in vitro* provided the theoretical basis for using H-BMSCs in the treatment DDD *in vivo*. To study whether HP could improve the therapeutic effect of BMSCs to IVD degeneration, we assessed the transplanted BMSC survival and migration after 4 weeks (Figure 6), DHI (Figure 5) and matrix-associated protein collagen II, aggrecan, and MMP-13 (Figure 7) in IVDs. BMSC transplantation can effectively treat DDD, and this result is consistent with previous studies [8–11]. The HP could enhance the BMSC treatment effect to DDD, which is consistent with the results *in vitro*. HP can enhance the capacity of BMSCs to repair DDD by increasing implanted cell survival and migration.

IVD degeneration is an inevitable consequence of aging and disc pressure, and its associated problems such as low back pain are severe health problems with a large socioeconomic burden [39]. Cell therapy is a new hopeful therapy for this disease [40, 41]. Various kinds of mesenchymal stem cells have been used to repair the DDD and achieve good results, such as the cells from the bone marrow [8–11], fat [42], umbilical cord blood [43], Wharton's jelly [44], olfactory stem cells [45], and induced pluripotent stem cells [46]. BMSCs received widespread attention for its easy to obtain, pluripotent, and multipotent abilities [8–11]. The research about the comparisons between the stem cells and differentiated cells to treat the DDD is limited and controversial, which needs future research. The differentiated cells may be superior in producing more ECM and be inferior in antidamage ability [47, 48]. However, there are many barriers that need to be solved in cell therapy. Injection into IVDs will speed up degeneration of IVDs. In the process of cell transplantation, it is necessary to use a needle to penetrate the fibrous rings and enter into the nucleus pulposus, which leads to destruction of the structural integrity of IVDs. Carragee's study confirmed that IVD degeneration significantly accelerated in patients who had undergone discography [49]. Vadala et al. found that BMSCs may leak out of the nucleus and occur undesirable osteophyte [50]. To minimize these complications, a 33-gauge microinjector was used and cell transplantation for each IVD was at least 5 min in this study. On one hand, the minimal needle was used to reduce disc degeneration caused by the cell transplantation itself; on the other hand, the injection time was at least 5 min to reduce cell leakage. A key barrier restricting cell transplantation application in DDD treatment is the special hypertonic and hypoxic environment in IVDs. The implanted cells may suffer from overt cell loss, cell activity, and differentiation decreased in this harsh, nutrient-poor environment [51, 52]. The application of HP for transplanted stem cell therapies has been reported for many diseases in recent years, such as infarcted heart [26] and ischemic stroke [27, 53]. Considering the special IVD environment with biomechanical stress and hypoxia, we demonstrated that HP could significantly enhance the capacity of BMSCs to repair DDD and provided an effective way to solve the overt cell loss, cell activity, and differentiation decreased in the cell transplantation of DDD treatment.

It is controversial how to accurately determine the BMSC differentiated into NP- or AF-like cells *in vivo* so far. There are only limited direct researches about the cell differentiation in the IVD in the published paper from PubMed [8–11]. In these papers, there were no standard and exact indicators to determine the differentiation of BMSCs into IVD. To study the differentiation of transplanted BMSCs into IVD tissue, we had tried to use immunohistochemistry to detect the expression of GFP, collagen II, and aggrecan in transplanted BMSCs by using antibody collagen II and anti-rat aggrecan (Supplementary Fig. 1). From the immunohistochemistry result, we found that the transplanted BMSCs could express the collagen II and aggrecan. We also found that all of the NP cells and the extracellular matrix in the nucleus pulposus region were stained by immunohistochemistry collagen II and aggrecan, and the transplanted BMSCs surround the positive extracellular matrix. All these dates may lead to the false positive of transplanted BMSCs. The research about the differentiation of BMSCs into IVD cell/tissue needs standard and exact indicator, which is a direction of our future research.

CoCl_2_ is a classical chemical hypoxia simulator and widely used because of the advantages of simple use and easy, precise control of treatment conditions [54–56]. The mechanism of its hypoxia simulation is the replacement of Fe^2+^ by Co^2+^ in hemoglobin, changing hemoglobin to a deoxidized state. In this state, the cells “feel” hypoxic in a normoxic environment [57]. It is easy to control the experimental conditions with CoCl_2_ *in vitro*, though there are some limitations. There is no evidence about the role of CoCl_2_ accumulation in cells. Therefore, we repeatedly washed with normal culture medium to minimize the influence of residual CoCl_2_ in the culture medium.The mechanism of CoCl_2_ is that Co^2+^ increases the expression of HIF and CXCR4 [26–28, 58–60]. CXCR4 is considered a key factor in migration of BMSCs [61]. Cell culture conditions could decrease the migration ability of BMSCs via downregulating the CXCR4 expression [62]. The fresh isolated BMSCs can migrate to the damaged tissue, while the migration ability will decrease significantly after BMSC culture *in vitro* after 24 h. HP could promote the migration of BMSCs that have been demonstrated in some researches [63–67]. In this study, we confirmed that HP induced by CoCl_2_ could promote migration, homing, and colonization of BMSCs via the pathway of CXCR4 and HIF-1 *in vitro*.

The optimal HP condition should have a slight effect on cell viability and improve migration and cell tolerance to damage significantly. Therefore, according to the results of the cell proliferation and apoptosis, 100 *μ*M CoCl_2_ cultured for 24 h was selected. The effects of CoCl_2_ were concentration dependent [60]. Long-term cell culture time may lead to cell differentiation, senescence, and other problems [68, 69]. Hypoxia may lead to differentiation of BMSCs [70] and affect cell activity and function via pathways of HIF [71, 72]. It may be more appropriate to select a higher concentration of CoCl_2_ for a shorter culture time, without significantly reducing cell activity and increasing apoptosis. HP can lead to the adaptability change in BMSCs. Although the mechanism was unclear, the adaptability change in BMSCs can produce stronger adaptability and resistance to the subsequent strong and harmful stimulation. HP can promote BMSC migration via the HIF-1*α* and CXCR4 pathways and decrease apoptosis by way of caspase-3 and bcl-2, which was confirmed in this study.

## 5. Conclusion

In conclusion, transplantation of H-BMSC retards IVD degeneration via enhancing implanted cell survival and migration in rats, which provides an effective way to improve the effects of BMSC transplantation in DDD treatment. However, the environment and degenerate model of IVD are different between rats and humans. Further studies should be performed about H-BMSC transplantation in clinical applications.

## Figures and Tables

**Table 1:** Sequences of primers used for RT-PCR.

Gene	Forward primer (5′-3′)	Reverse primers (5′-3′)
GAPDH	GCAAGTTCAACGGCACAG	GGCCCCTCCTGTTGTTATGG
HIF-1*α*	CACTGCACAGGCCACATTCAT	AAGCAGGTCATAGGCGGTTTC
CXCR4	TCCGTGGCTGACCTCCTCTT	CAGCTTCTCGGCCTCTGGC
bcl-2	TCCTTCCAGCCTGAGAGCAACC	CGACGGTAGCGACGAGAGAAG
Caspase-3	GCGGTATTGAGACAGACAGTGGAAC	GCGGTAGAGTAAGCATACAGGAAGT

**Table 2:** Effect of CoCl_2_ on the proliferation of BMSCs detected by CCK-8 assay.

Concentration/time	0 h	6 h	12 h	24 h	48 h
0 *μ*M	1.01 ± 0.06	1.11 ± 0.09	1.41 ± 0.18^#^	1.82 ± 0.19	2.6 ± 0.18^#^
10 *μ*M	1.00 ± 0.08	1.05 ± 0.09	1.30 ± 0.13^#^	1.56 ± 0.18	2.38 ± 0.10^#^
50 *μ*M	1.00 ± 0.09	0.95 ± 0.12	1.14 ± 0.18	1.30 ± 0.12^∗^	1.75 ± 0.16^∗^
100 *μ*M	1.01 ± 0.10	0.85 ± 0.14	0.98 ± 0.12^#^	1.15 ± 0.11	1.41 ± 0.11^#^
200 *μ*M	1.03 ± 0.09	0.80 ± 0.17	0.88 ± 0.15	0.95 ± 0.16^∗^	0.89 ± 0.13^∗^
300 *μ*M	1.01 ± 0.06	0.72 ± 0.10	0.58 ± 0.13	0.44 ± 0.28	0.24 ± 0.10

^∗^ *P* < 0.05 versus 100 *μ*M group, ^#^ *P* < 0.05 versus 24 h group (mean ± SD, *n* = 6).

## Supplementary Materials

Supplementary Materials Supplementary Figure 1: differentiation of BMSCs into IVD 4 weeks after transplantation (200×). A and E represent the cell nucleus image overlaid by DAPI. B and F show GFP-positive BMSCs. C shows collagen II-positive cells. G shows aggrecan-positive cells. D merging of A, B, and C. H merging of E, F, and G.
