# Supplementary material for: Transplantation of Hypoxic-Preconditioned Bone Mesenchymal Stem Cells Retards Intervertebral Disc Degeneration via Enhancing Implanted Cell Survival and Migration in Rats
Source: Stem Cells Int. 2018 Feb 14;2018:7564159. doi: 10.1155/2018/7564159 (PMC5832130; doi:10.1155/2018/7564159)
Supplement: Supplementary Materials — Supplementary Figure 1: differentiation of BMSCs into IVD 4 weeks after transplantation (200×). A and E represent the cell nucleus image overlaid by DAPI. B and F show GFP-positive BMSCs. C shows collagen II-positive cells. G shows aggrecan-positive cells. D merging of A, B, and C. H merging of E, F, and G. [file 7564159.f1.docx]

A


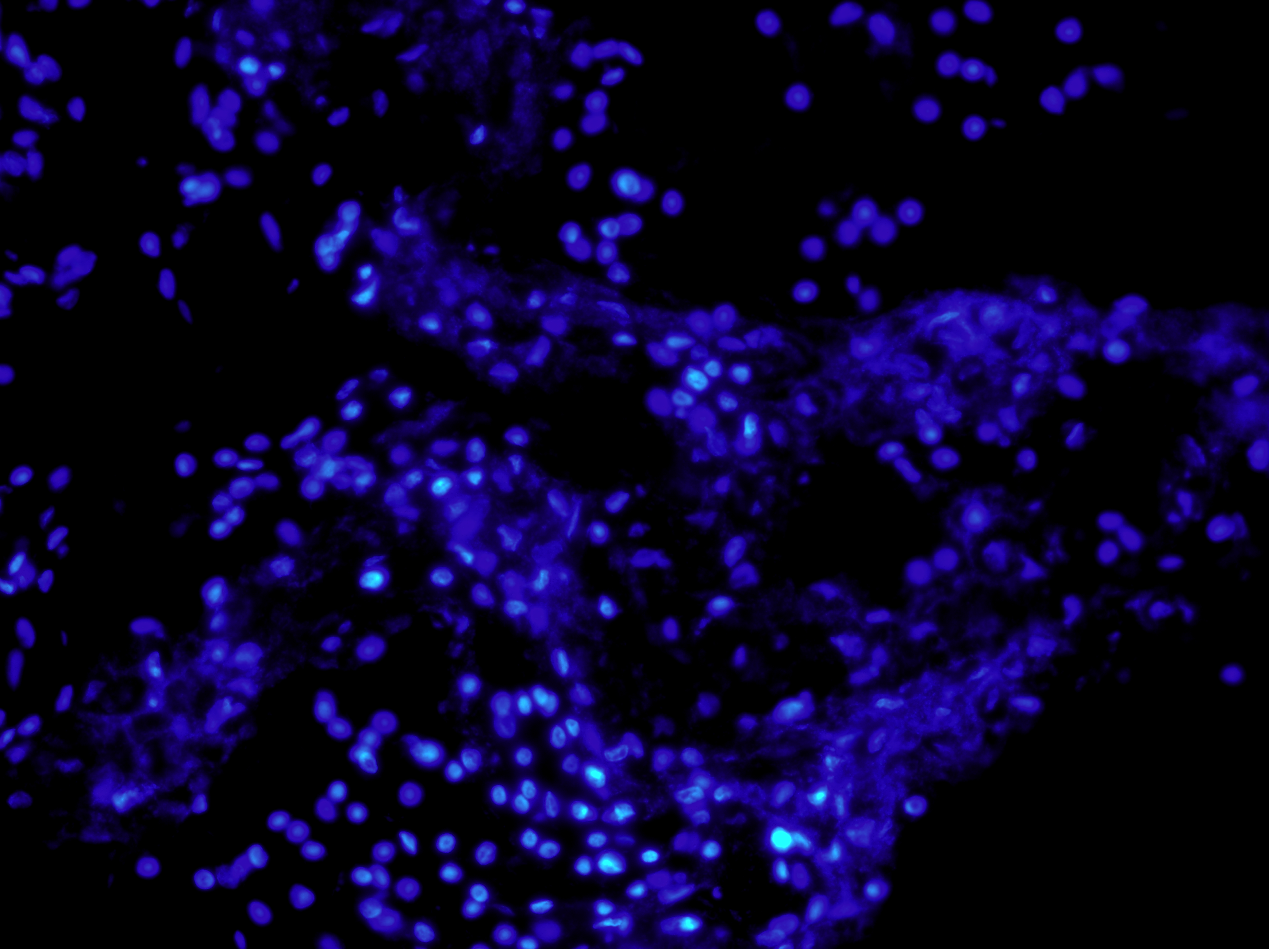


B


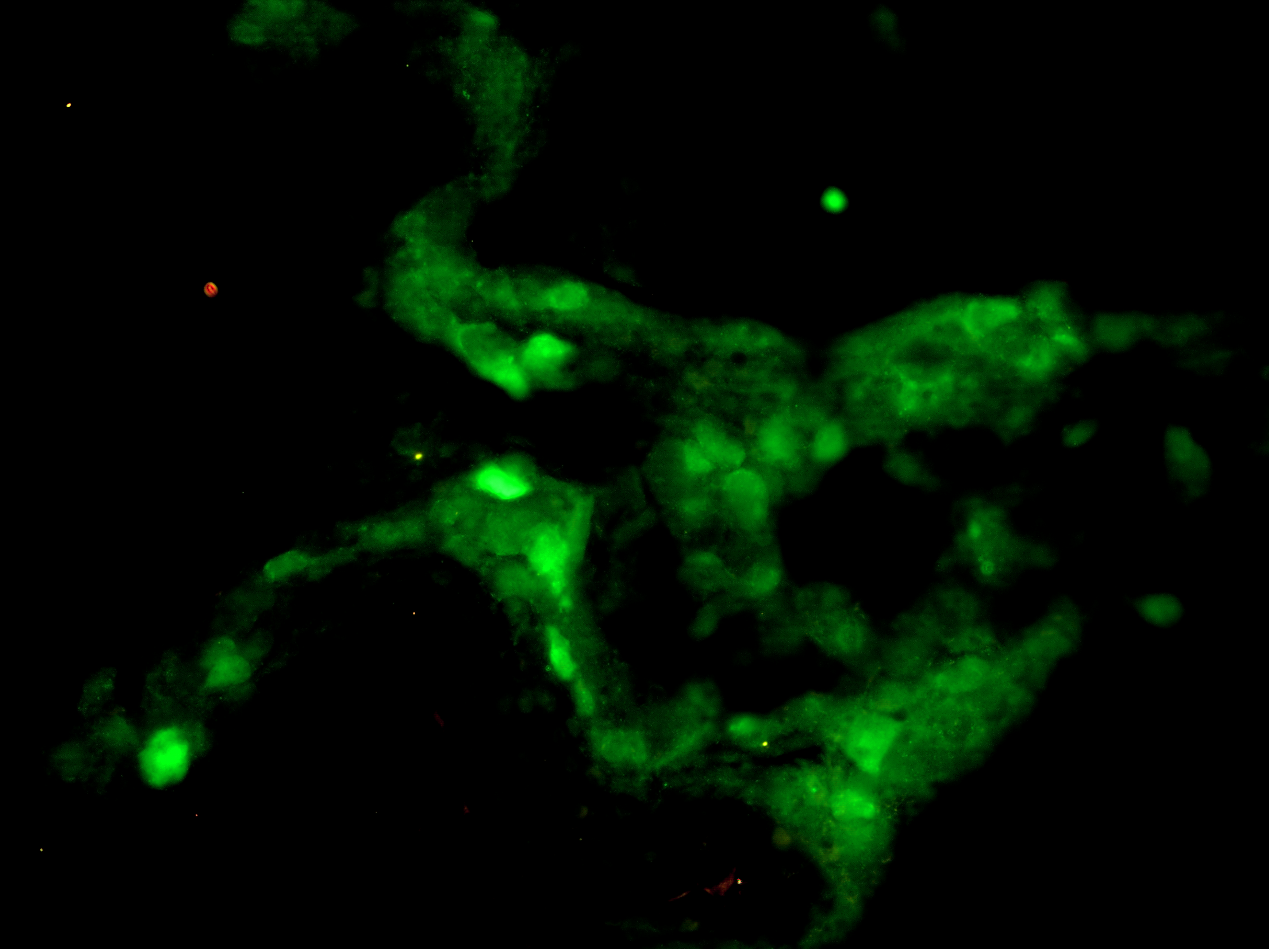


C


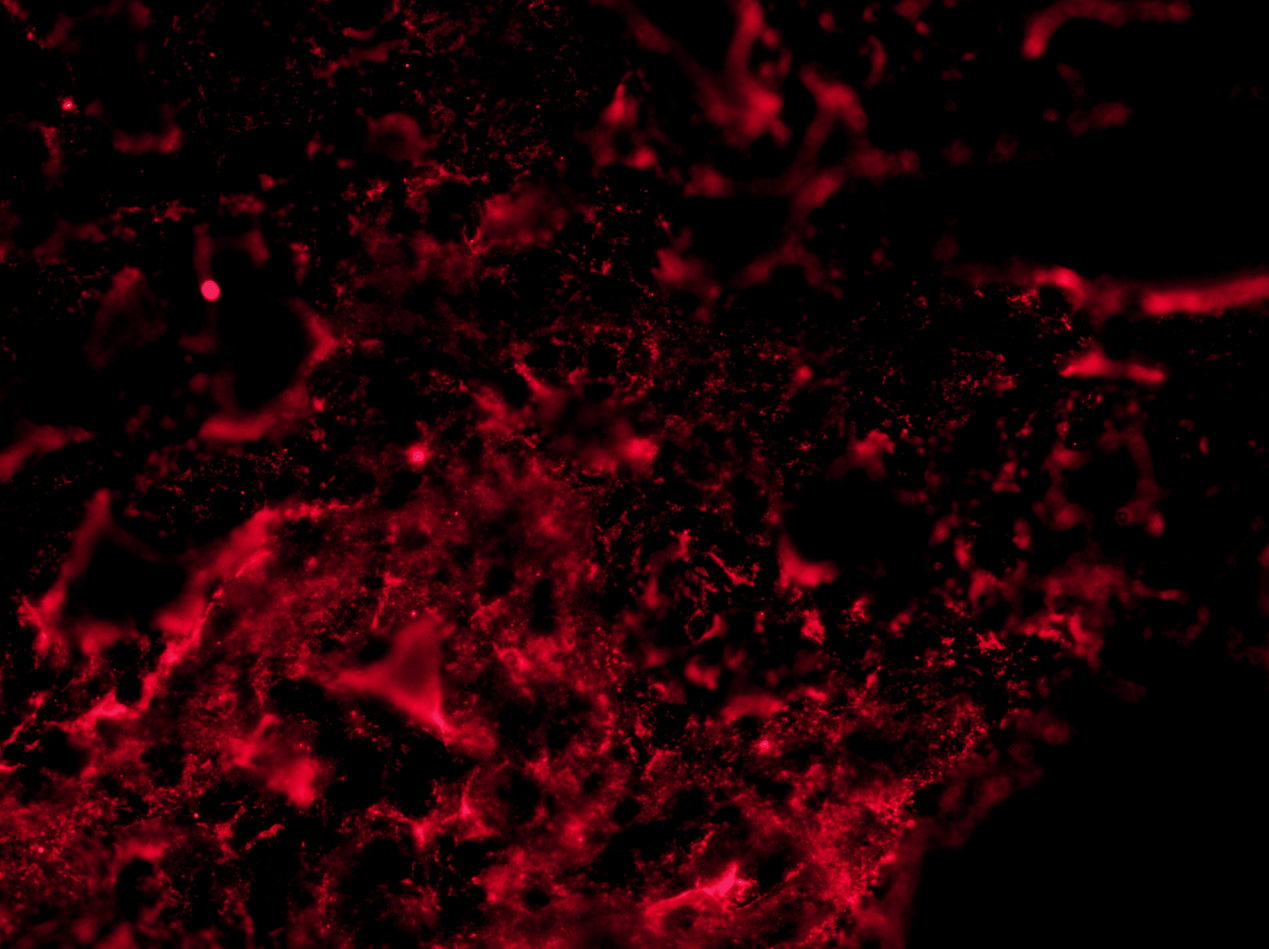


D

**
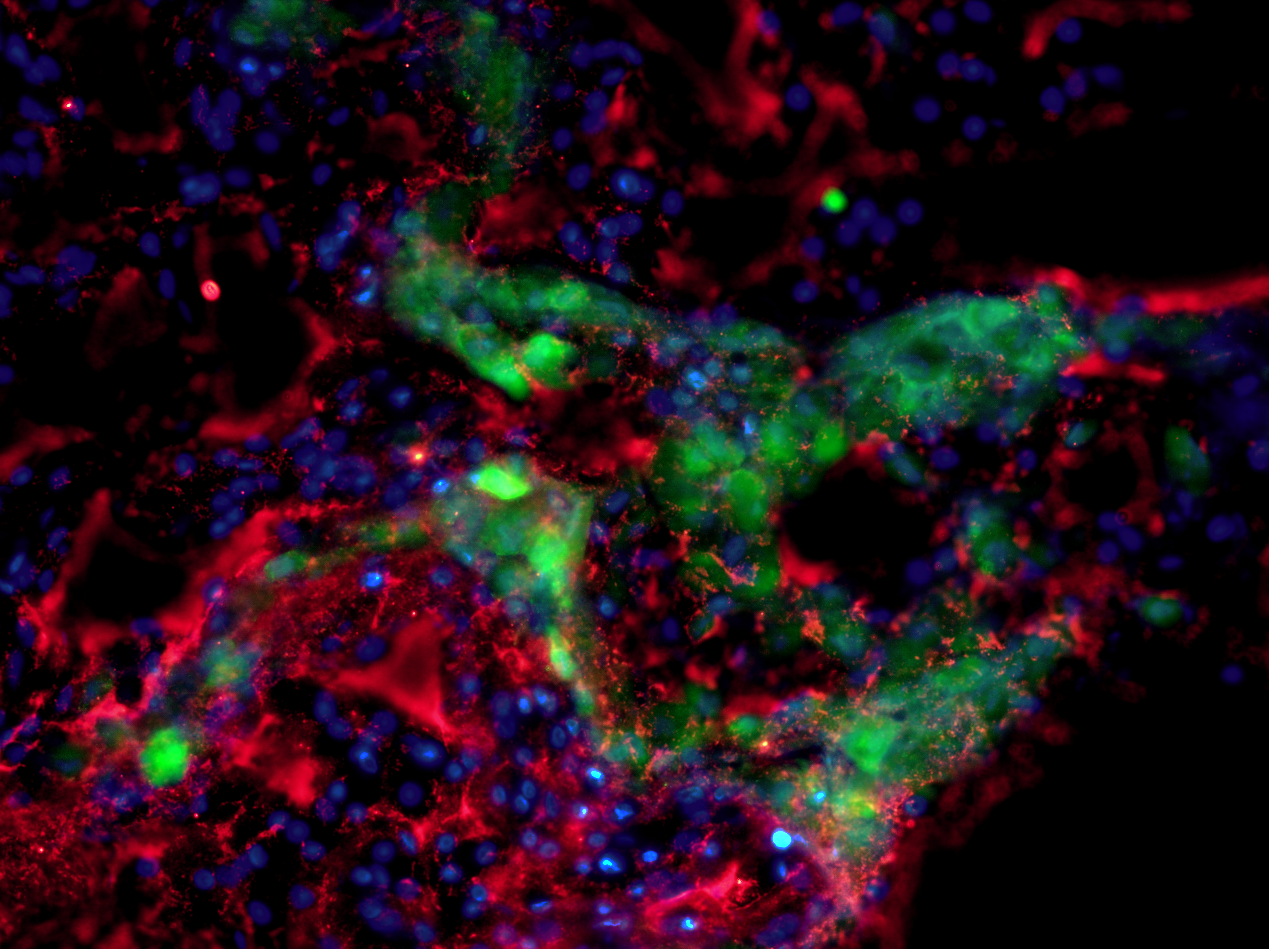
**

**E**

**
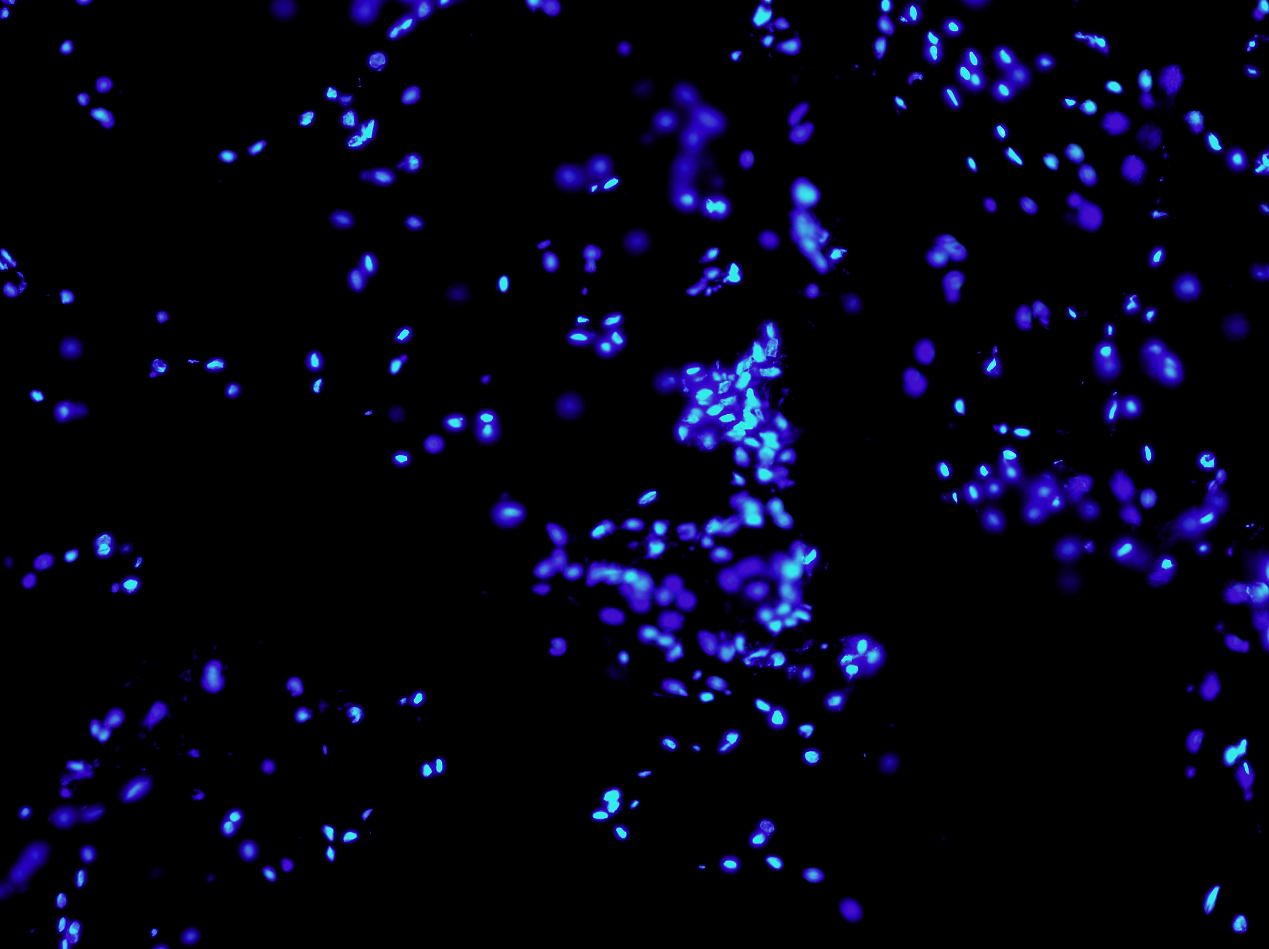
**

**F**

**
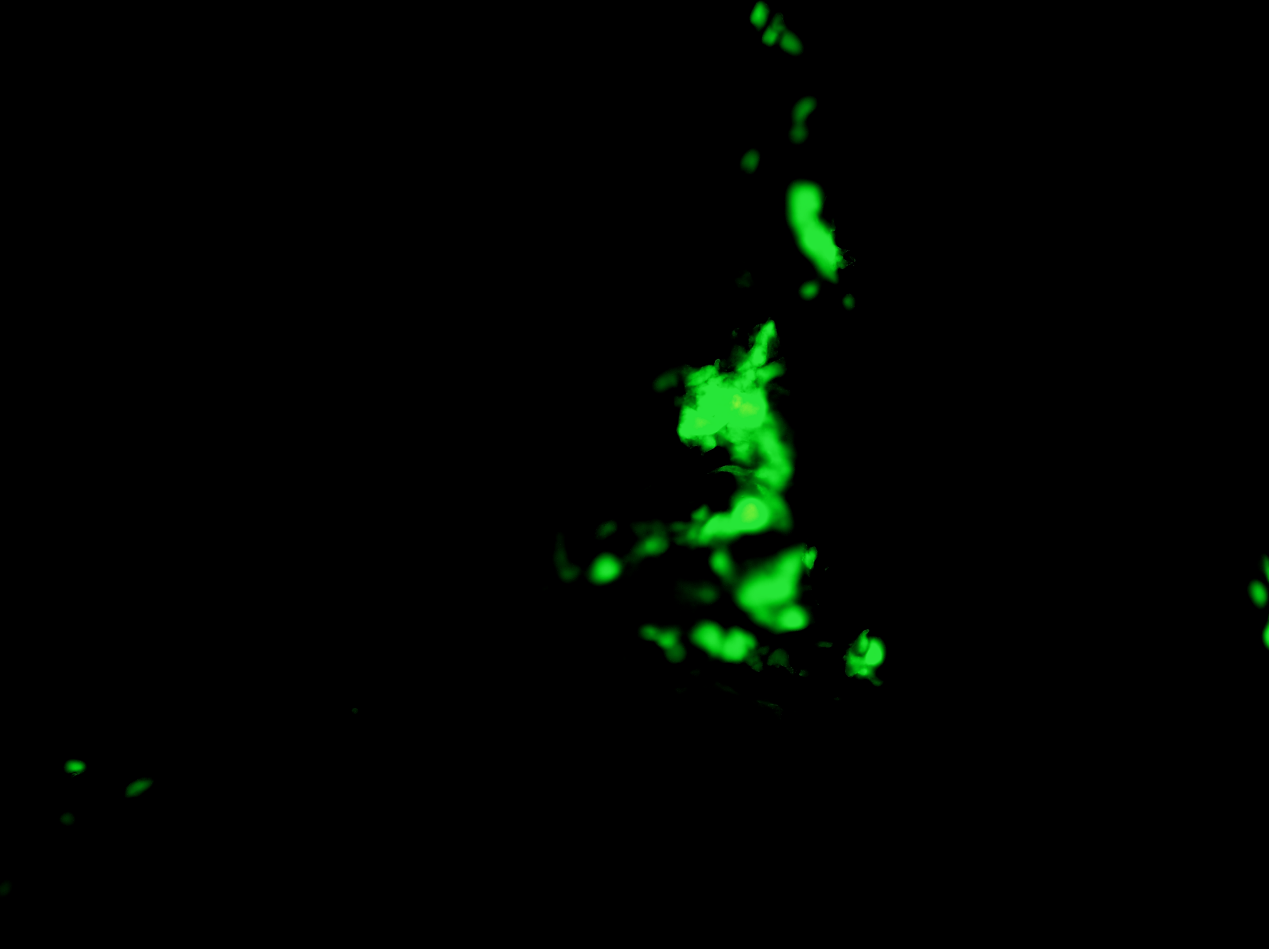
**

**G
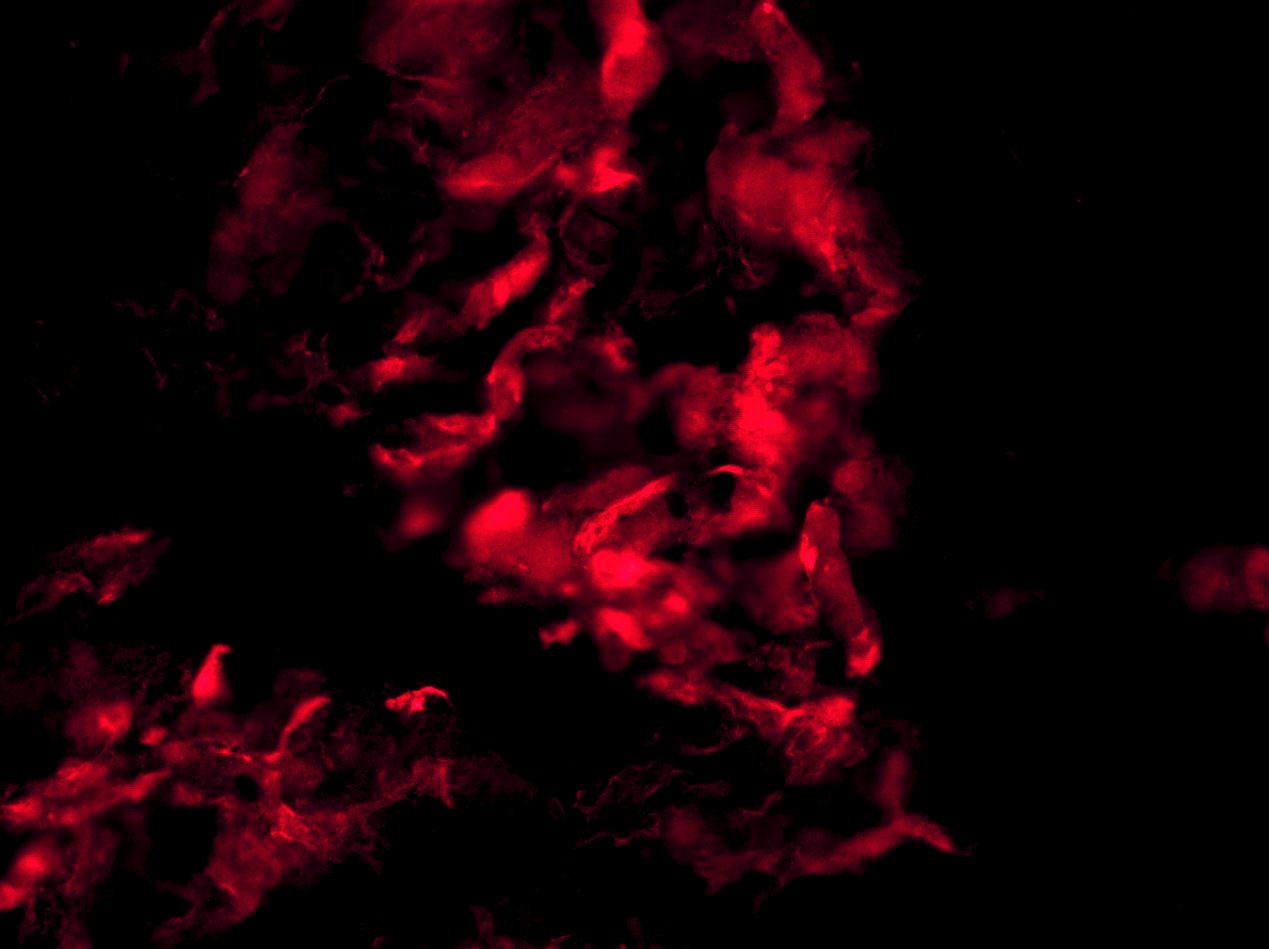
**

**H**

**
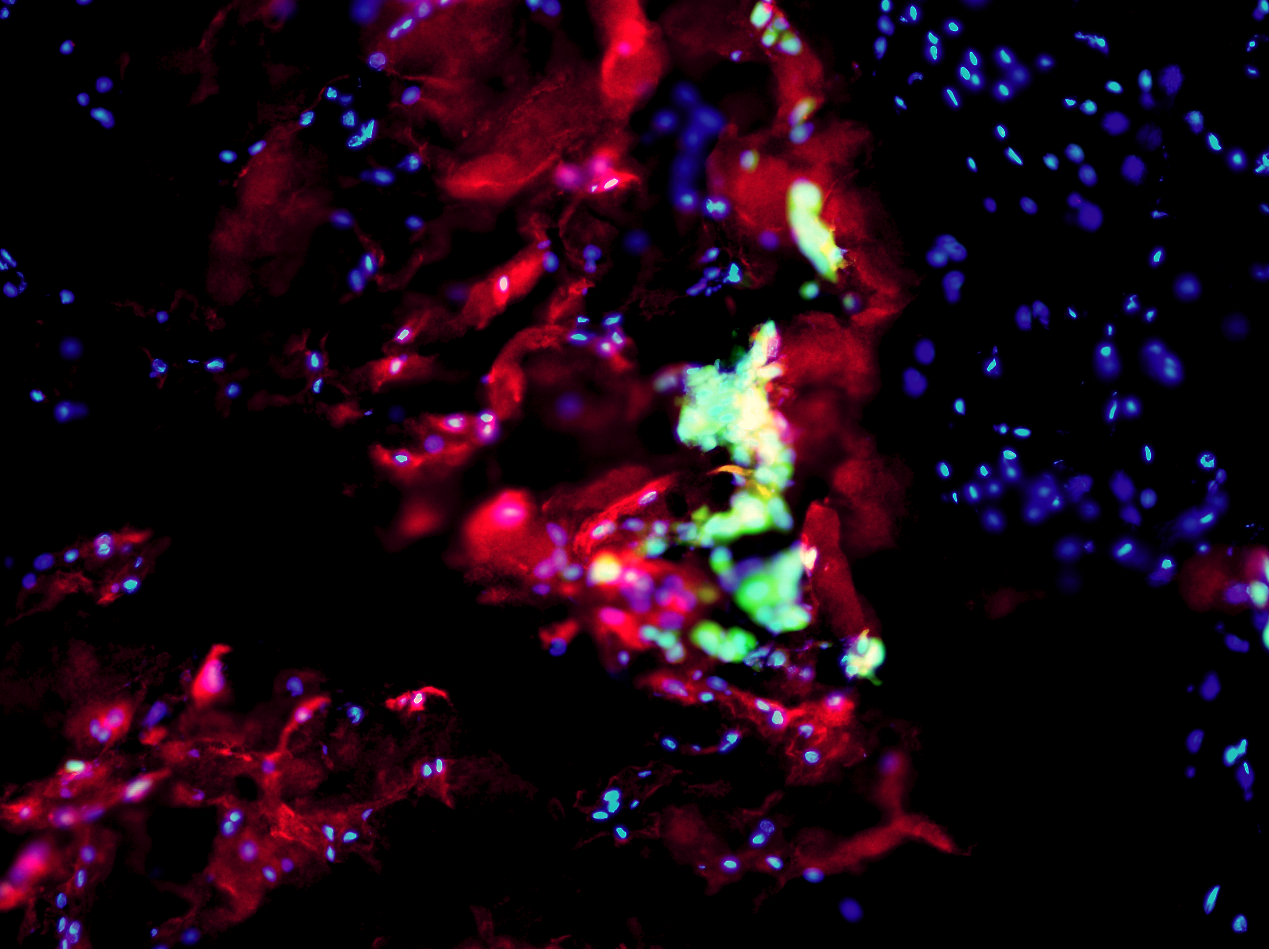
**

Supplementary figure. 1 Differentiation of BMSCs into IVD 4 weeks after transplantation (200×). A and E represent the cell nucleus image overlaid by DAPI. B and F show GFP-positive BMSCs. C show collagen II positive cells. G show aggrecan positive cells. D Merging of A, B and C. H Merging of E, F and G.
